# Vaccine Adjuvants in the Immunocompromised Host: Science, Safety, and Efficacy

**DOI:** 10.1111/tid.70053

**Published:** 2025-05-19

**Authors:** Haya Hayek, Lana Hasan, Justin Z. Amarin, Yasmeen Z. Qwaider, Olla Hamdan, Wanderson Rezende, Kevin C. Dee, James D. Chappell, Natasha B. Halasa

**Affiliations:** ^1^ Department of Pediatrics Vanderbilt University Medical Center Nashville Tennessee USA; ^2^ Department of Infectious Disease Cleveland Clinic Foundation Cleveland Ohio USA; ^3^ Epidemiology Doctoral Program School of Medicine Vanderbilt University Nashville Tennessee USA; ^4^ Department of Infectious Diseases Vanderbilt University Medical Center Nashville Tennessee USA

## Abstract

Vaccine adjuvants are essential for enhancing immune responses to vaccines, particularly in immunocompromised populations who typically demonstrate suboptimal responses to standard vaccination. This narrative review evaluates the safety and efficacy of approved and candidate adjuvants in immunocompromised hosts, with emphasis on solid organ and hematopoietic cell transplant recipients. We examine conventional aluminum‐based adjuvants alongside modern adjuvant systems such as AS01_B_, MF59, and AS04, analyzing their mechanisms of action and clinical applications. The review synthesizes current evidence on the safety profiles of approved adjuvanted vaccines in immunocompromised individuals and explores emerging adjuvant candidates, including saponin complexes and toll‐like receptor agonists. By examining factors that influence adjuvant immunogenicity and safety in these vulnerable populations, we identify critical knowledge gaps and future research priorities. This comprehensive analysis provides clinicians and researchers with an updated perspective on the rapidly evolving landscape of vaccine adjuvants and their specific applications in immunocompromised hosts.

**Abbreviations**
ACIP: Advisory Committee on Immunization Practices
AHQ‐II: alhydroxiquim‐II
ALF: Army Liposome Formulation
APC: antigen‐presenting cell
AST: American Society of Transplantation
CDC: Centers for Disease Control and Prevention
COVID‐19: coronavirus disease 2019
CpG: cytosine‐phosphate‐guanine
DAMP: danger‐associated molecular pattern
DC: dendritic cell
DTaP: diphtheria, tetanus, and acellular pertussis
FDA: Food and Drug Administration
GLA‐SE: glucopyranosyl lipid A in stable emulsion
HCT: hematopoietic cell transplant
HIV: human immunodeficiency virus
HPV: human papillomavirus
IC: immunocompromised
ICMR: Indian Council of Medical Research
IMQ: imidazoquinoline
ISCOM: immune‐stimulating complexes
LPS: lipopolysaccharide
MAPKs: mitogen‐activated protein kinases
MOA: mechanism of action
MPL: monophosphoryl lipid A
NF‐κB: nuclear factor kappa B
NIAID: National Institute of Allergy and Infectious Diseases
NIV: National Institute of Virology
PAMP: pathogen‐associated molecular pattern
PCV: pneumococcal conjugate vaccine
PLWH: people living with HIV
PPSV23: pneumococcal polysaccharide vaccine 23
PRR: pattern recognition receptors
RSV: respiratory syncytial virus
RZV: recombinant zoster vaccine
SARS‐CoV‐2: severe acute respiratory syndrome coronavirus 2
SOT: solid organ transplant
Td: tetanus–diphtheria
TLR: toll‐like receptor
US: United States
VE: vaccine efficacy
WHO: World Health Organization

## 1 Introduction

Vaccine adjuvants are substances that augment the immune response to vaccine antigens, thereby amplifying the immunogenicity of vaccines. They are crucial for achieving robust protective immunity against diverse pathogens and may facilitate antigen‐sparing or reduce the number of vaccine doses required. Adjuvant use is pivotal in modern vaccine development. Adjuvants such as aluminum salts and water‐in‐oil emulsions have been employed since the 1920s and 1930s, respectively, with novel adjuvants continually emerging since that time. The dynamic global landscape of emerging and re‐emerging pathogens, coupled with the increasing prevalence of immunocompromised (IC) populations, reinforces the imperative need for ongoing innovation and research into adjuvants.

While the mechanisms of adjuvants are not fully understood, they commonly enhance vaccine efficacy by activating innate immune responses. They often target pattern recognition receptors (PRRs), such as toll‐like receptors (TLRs), which play a pivotal role in recognizing pathogen‐associated molecular patterns (PAMPs) and danger‐associated molecular patterns (DAMPs) [1]. Upon activation, these receptors initiate signaling pathways that induce antigen‐presenting cells (APCs) and the subsequent humoral and cell‐mediated responses. This cascade augments the immediate immune response and aids in establishing long‐lasting immunity by promoting the development of memory immune cells [2]. However, the specificity of these responses can vary significantly based on the adjuvant composition and the host's immune status, necessitating a tailored approach in vaccine formulation.

Effective adjuvants are especially important for IC individuals, such as organ transplant recipients, those living with human immunodeficiency virus (HIV) or cancer, and those undergoing chronic immunosuppression for inflammatory disorders. These groups are more susceptible to infections, including routine, opportunistic, and emerging pathogens, and often respond suboptimally to standard vaccines. They are often at heightened risk for such infections due to diminished immune memory and inadequate vaccine responses. The extent of their suboptimal vaccine responses varies significantly depending on the type of immunocompromising condition. For example, people with well‐controlled HIV may mount relatively good vaccine responses (often with seroconversion >60% compared with healthy individuals), whereas those receiving B‐cell‐depleting therapies or undergoing solid organ or hematopoietic cell transplantation may exhibit poor vaccine responses (often with seroconversion <40% compared with healthy individuals) [3]. This wide spectrum of immune deficits underscores the critical need for potent adjuvanted vaccines tailored to these subpopulations. Recent outbreaks of H5N1 avian influenza in the United States (US) and internationally, including documented zoonotic transmissions from avian and bovine sources, exemplify the need for vaccines incorporating adjuvants to enhance immunogenicity against novel influenza viruses [4, 5].

Development of improved adjuvanted vaccines targeted at IC individuals has become a priority to help elicit more robust immune responses and provide better protection. Clinically available adjuvants such as MF59 and AS04 have demonstrated efficacy and safety within this demographic, supporting their use in vaccine formulations [6]. Despite these benefits, few adjuvants are currently approved for use in IC individuals, and concerns over safety and efficacy persist [7]. Numerous studies affirm that adjuvanted vaccines are generally well‐tolerated and do not significantly elevate the risk of serious adverse events compared to nonadjuvanted vaccines [8]. However, systemic immunopathologic reactions induced by adjuvanted vaccines may present insidiously, posing a significant challenge to the establishment of vaccine safety [9]. Furthermore, it is reasonable to anticipate differences in the efficacy and safety of adjuvanted vaccines across the spectrum of immunocompromising conditions (e.g., primary and secondary immunodeficiencies, organ and stem cell transplantation, autoimmunity) and age groups, highlighting the challenge of generalizing findings from the limited available data to the broader IC population. Heterogeneous mechanisms of immune dysregulation likely foster response variation to the same adjuvant, while the effect profiles of different adjuvants will vary in relation to the specific immunocompromising condition.

In this narrative review, we discuss the mechanisms of action (MOA), historical development, and safety profiles of both traditional and modern vaccine adjuvants. We focus on clinically available adjuvants currently used for IC individuals, examining their immunogenicity, safety, and specific limitations associated with their use. We identify areas where further innovation in adjuvant research is critically needed and present ongoing debates regarding the balance between effectiveness and safety in these vulnerable patients. Our discussion encompasses historically significant adjuvants, those currently approved with relevance to IC populations, key candidates in development representing diverse mechanistic classes, and adjuvants illustrative of specific safety or efficacy considerations. We synthesized literature identified through broad searches on these adjuvants and prioritized data on various IC subpopulations, particularly solid organ transplant (SOT) and hematopoietic cell transplant (HCT) recipients, without formally restricting our searches to these subpopulations.

## 2 Approved Adjuvants

Several adjuvants have received regulatory approval and are currently used in licensed vaccines. Table 1 summarizes the major approved adjuvants and current vaccine applications, accompanied by key clinical recommendations for immunocompromised populations. In the following sections, we discuss approved adjuvants in detail, examining development, mechanisms of action, and specific considerations for immunocompromised populations.

**TABLE 1:** Currently approved vaccine adjuvants: Clinical applications and use in immunocompromised populations.

Adjuvant	Vaccine applications	Safety	Efficacy	Key recommendations	Additional notes
Aluminum salts	DAPTACEL/INFANRIX (DTaP); HAVRIX (hepatitis A); TWINRIX (hepatitis A/B); ENGERIX‐B (hepatitis B); Prevenar 13/20 (PCV)	Studied in HCT/SOT recipients; generally considered safe [16] HAVRIX is safe in liver and kidney transplant recipients [17] Td vaccine is safe in kidney transplant recipients [19]	HAVRIX elicited 97% seroconversion in liver and 72% in kidney transplant recipients after two doses [17] Hepatitis B vaccines evoked lower immunogenicity vs. healthy controls [18]	Hepatitis A/B vaccines recommended pre‐transplantation; multiple doses may be needed post‐transplantation ACIP recommends specific PCV20/PCV15 schedules for HCT/SOT recipients [20]	Oldest, most widely used adjuvant class Lower vaccine immunogenicity vs. healthy controls often necessitates modified schedules or higher doses in immunocompromised hosts
CpG 1018	HEPLISAV‐B (hepatitis B)	Demonstrated safety in transplant recipients, liver cirrhosis patients, immunosuppressive biologic recipients, and people living with HIV [27, 28, 29, 30]	Demonstrated immunogenicity in transplant recipients, liver cirrhosis patients, immunosuppressive biologic recipients, and people living with HIV, and is often more immunogenic than its aluminum‐adjuvanted counterpart [27, 28, 29, 30]	HEPLISAV‐B is recommended by ACIP for adults ≥18 years at risk of hepatitis B, including those with end‐stage liver disease or HIV [31]	TLR9 agonist HEPLISAV‐B requires fewer doses than ENGERIX‐B in general population trials [25, 26]
MF59	FLUAD (pre‐pandemic H1N1 and 2009 H1N1 pandemic influenza); Focetria and Celtura (pandemic influenza)	Studied in the SOT population, with no significant differences in allograft rejection between standard and MF59‐adjuvanted vaccines [39]	Considered effective based on clinical recommendations; specific comparative efficacy data are less extensive	FLUAD is approved for adults ≥65 years in the United States For 2024–2025 season, ACIP stated that FLUAD may be used in SOT recipients aged 18–64 years on immunosuppression regimens [36]	Oil‐in‐water (squalene) emulsion Historical concerns regarding Gulf War syndrome are unsubstantiated [35, 37]
AS04	Fendrix (hepatitis B); formerly in Cervarix (HPV)	Fendrix is designed for patients with renal disease and those on hemodialysis, implying acceptable safety in this subpopulation	Fendrix elicits higher antibody titers vs. alum‐only vaccines in the target population [40]	Fendrix is recommended for renal patients in Europe and is not available or marketed in the United States	MPL (TLR4 agonist) adsorbed on aluminum salts Cervarix was discontinued in the United States due to marketing decisions, not safety issues [42]
AS03	Previously in Prepandrix (influenza H5N1) and Pandemrix (influenza H1N1)	Multiple studies affirm safety in immunocompromised individuals [48]	Multiple studies affirm efficacy in immunocompromised individuals [48]	No current routine recommendations Potential for use in future influenza pandemic responses [46]	Oil‐in‐water emulsion supplemented with vitamin E Historical association of Pandemrix with narcolepsy in genetically susceptible (HLA‐DQB1*06:02) individuals [47, 48]
AS01_B_	SHINGRIX (herpes zoster)	Safe for HIV, hematologic malignancies, oncologic conditions, and SOT/HCT [52, 54–58, 60]	Generally efficacious Overall vaccine efficacy of 68% in autologous HCT recipients, though efficacy was lower in post‐transplant patients ≥50 years of age [61]	ACIP recommends two doses of SHINGRIX for immunocompromised individuals ≥19 years old [63] AST recommends pre‐transplant immunization [59]	MPL and QS‐21 Significant but transient reactogenicity Post‐transplant efficacy data are limited
AHQ‐II	Formerly in BBV152/COVAXIN (SARS‐CoV‐2)	Specific safety data lacking in immunocompromised individuals [72]	Lower efficacy among immunocompromised individuals [73]	BBV152/COVAXIN was provisionally recommended by the WHO for immunocompromised individuals [72]	IMQ (TLR7/8) adsorbed onto alum Use was suspended due to effectiveness concerns [74]
Virosomes	Inflexal V (influenza), Epaxal (hepatitis A)	Inflexal V shows minimal side effects in immunocompromised individuals [76, 77]	Inflexal V shows strong immune responses in immunocompromised individuals [76, 77]	No specific recommendations	Lipid bilayer delivery system mimicking virus structure Promising platform for tailored vaccination strategies [79]
RC‐529	Formerly in Supervax (hepatitis B)	Not specifically studied in immunocompromised individuals Well‐tolerated in healthy adults [80]	Not specifically studied in immunocompromised individuals More immunogenic in healthy adults than standard vaccines [80]	Supervax is no longer in use [82]	Synthetic lipid A mimetic (TLR4 agonist) Developed as a less toxic alternative to MPL

*Note*: This table summarizes major vaccine adjuvants, their current vaccine applications, documented studies in immunocompromised hosts, and key clinical recommendations or safety considerations.

Abbreviations: ACIP, Advisory Committee on Immunization Practices; AHQ‐II, alhydroxiquim‐II; AST, American Society of Transplantation; DTaP, diphtheria, tetanus, and acellular pertussis; HCT, hematopoietic cell transplant; HIV, human immunodeficiency virus; HPV, human papillomavirus; IMQ, imidazoquinoline; MPL, 3‐*O*‐desacyl‐4′‐monophosphoryl lipid A; PCV, pneumococcal conjugate vaccine; SARS‐CoV‐2, severe acute respiratory syndrome coronavirus 2; SOT, solid organ transplant; Td, tetanus–diphtheria; TLR, toll‐like receptor; WHO, World Health Organization.

### 2.1 Aluminum Salts

Aluminum‐based adjuvants are among the oldest and most widely used in vaccines. The adjuvanticity of potassium aluminum sulfate, or “alum,” was first described in the 1920s [10]. Over time, traditional alum has been supplanted by more consistent formulations (batch‐to‐batch reproducibility) such as aluminum hydroxide and aluminum phosphate [10, 11]. These adjuvants are components of numerous vaccines approved by the US Food and Drug Administration (FDA), including diphtheria, tetanus, and pertussis vaccines (DAPTACEL by Sanofi Pasteur, INFANRIX by GSK, Adacel by Sanofi Pasteur, BOOSTRIX by GSK); combined hepatitis A and hepatitis B vaccine (TWINRIX by GSK); hepatitis B vaccine (ENGERIX‐B by GSK); and pneumococcal vaccines (PREVNAR 13 and 20 by Pfizer), among others [12]. The primary immunostimulatory mechanism of aluminum‐based adjuvants was classically hypothesized to be prolonged antigen presentation due to limited tissue solubility (“depot effect”) [13]. However, recent studies suggest that these adjuvants induce local inflammation, enhance dendritic cell activation, and stimulate the NLRP3 inflammasome pathway [14, 15].

Many aluminum‐adjuvanted vaccines are recommended for use in IC individuals, including HCT and SOT recipients [16]. To ensure optimal efficacy, aluminum‐adjuvanted vaccines should ideally be administered before solid‐organ transplantation, as multiple doses are required if vaccination occurs post‐transplant due to the varying net state of immunosuppression. Stark et al. evaluated the immunogenicity and safety of the hepatitis A vaccine, HAVRIX GSK (adjuvanted with aluminum hydroxide), in liver and kidney transplant recipients. They found that 41% of liver and 24% of kidney recipients seroconverted after one dose. After two doses, these proportions increased to 97% and 72%, respectively. In contrast, control groups achieved seroconversion proportions of 90% after one dose and 100% after two doses. The vaccine was found to be safe across all groups [17]. Similarly, aluminum‐adjuvanted hepatitis B vaccines (ENGERIX‐B, TWINRIX, and RECOMBIVAX HB [Merck & Co.]) are also recommended for transplant recipients, though they may exhibit lower immunogenicity compared with healthy controls [18]. Tetanus–diphtheria (Td) vaccine was also safe in kidney transplant recipients in a study by Enke et al. [19].

In 2023, the US Advisory Committee on Immunization Practices (ACIP) updated its recommendation for use of pneumococcal conjugate vaccines (PCVs), formulated with aluminum salts in the IC population. For HCT recipients, the guidelines recommend initiating vaccination 3–6 months post‐transplant with either three doses of PCV20 given 4 weeks apart followed by a fourth dose ≥6 months after the third dose or ≥12 months post‐transplant (whichever is later), or alternatively, three doses of PCV15 given 4 weeks apart followed by the adjuvant‐free pneumococcal polysaccharide vaccine 23 (PPSV23) at ≥12 months post‐transplant. SOT recipients should receive either a single dose of PCV20 or PCV15 followed by PPSV23 at least 1 year later [20].

Minimal safety concerns are associated with aluminum‐based adjuvants in the general population. However, a 2022 observational study reported a possible association between aluminum‐adjuvanted vaccine exposure and the development of persistent asthma in childhood [21]. The Centers for Disease Control and Prevention (CDC) does not recommend changes to current vaccination guidelines, but further studies are needed to evaluate this potential association.

### 2.2 CpG 1018

Cytosine‐phosphate‐guanine (CpG)‐containing oligonucleotide sequence, CpG 1018, was first studied in 1995 [22]. It is known as a potent stimulator of TLR9, leading to the activation of APCs and TLR9‐expressing B cells [23]. In 2017, a CpG 1018‐adjuvanted hepatitis B vaccine (HEPLISAV‐B; Dynavax Technologies) was FDA‐approved [24]. In clinical trials, HEPLISAV‐B elicited a protective response with fewer doses compared to the aluminum‐adjuvanted vaccine, ENGERIX‐B [25, 26]. HEPLISAV‐B demonstrated safety and immunogenicity in diverse IC individuals, including transplant recipients, those with liver cirrhosis, recipients of immunosuppressive biologics, and people living with HIV (PLWH) [27, 28, 29, 30]. In 2018, the ACIP recommended HEPLISAV‐B for individuals 18 years of age and older at risk of contracting hepatitis B, including those with end‐stage liver disease and PLWH [31].

### 2.3 MF59

MF59 (Novartis) is an oil‐in‐water emulsion containing the naturally occurring oil, squalene, stabilized in a citric acid buffer by the nonionic surfactants polysorbate 80 and Span 85 [6]. Although the exact MOA of MF59 has not yet been fully elucidated, MF59 has a short half‐life and thus does not depend on long‐term deposition at the site of vaccination [32, 33]. MF59 has been shown to elicit strong humoral (IgG) and cell‐mediated responses as well as to induce monocytes, macrophages, and dendritic cells (DCs) to secrete chemokines, which in turn recruit more leukocytic uptake of vaccine antigen [34]. Additionally, immune cells, namely neutrophils and monocytes, are induced to differentiate into APCs, which migrate to lymph nodes and prompt an adaptive immune response [34, 35].

MF59 was developed in the late 1980s, and the first clinical trial of an MF59‐adjuvanted vaccine was conducted in 1992. MF59 was approved in 1997 in Europe for inclusion in influenza vaccines (FLUAD [pre‐pandemic H1N1 and 2009 H1N1 pandemic influenza] and Focetria and Celtura for 2009 H1N1 pandemic influenza; CSL Seqirus). In the United States, FLUAD is only approved for adults 65 years and older. However, the ACIP stated that in the 2024–2025 influenza season, SOT recipients aged 18 through 64 years who are receiving immunosuppressive medication regimens may receive adjuvanted inactivated influenza vaccines as an acceptable option [36].

Claims that MF59‐adjuvanted vaccines were linked to Gulf War syndrome due to the development of squalene‐specific antibodies have not been substantiated [35, 37]. Meta‐analyses and controlled clinical trials have demonstrated that MF59‐adjuvanted vaccine use does not increase the incidence of autoimmune syndromes, chronic diseases, neurological conditions, or death [35, 38]. MF59‐adjuvanted influenza vaccines have been studied in the SOT population, with no significant differences in allograft rejection reported between patients receiving standard and adjuvanted vaccines [39].

### 2.4 AS04

AS04 (GSK) is a modified, detoxified form of *Salmonella minnesota* lipopolysaccharide (LPS) consisting of 3‐*O*‐desacyl‐4′‐monophosphoryl lipid A (MPL) adsorbed on aluminum salts [40]. Alum‐adsorbed MPL stimulates the immune system by interacting with TLR4, which activates nuclear factor kappa B (NF‐κB) signaling pathways to induce the production of proinflammatory cytokines and chemokines. APCs, such as monocytes and DCs, are thereby recruited to the site of vaccination and draining lymph nodes and interact with antigen‐specific T and B cells to elicit robust cellular and humoral immune responses [34, 41].

In 2009, AS04 was approved for use in the human papillomavirus (HPV) vaccine, Cervarix (GSK). Large population‐based database studies of Cervarix showed an acceptable safety profile in young females and males [42]. However, in 2016, Cervarix marketing in the United States was discontinued due to low demand. Currently, AS04 is incorporated as an adjuvant in Fendrix (GSK), a hepatitis B vaccine administered in Europe and specifically designed for patients with renal disease and those on hemodialysis. The addition of AS04 to both vaccines resulted in higher antibody titers to the same antigens adjuvanted with alum alone, demonstrating the added benefit of the MPL in people [40].

### 2.5 AS03

AS03, also developed by GSK, is an oil‐in‐water emulsion containing polysorbate 80 and squalene supplemented with DL‐α‐tocopherol (vitamin E), a potent immunostimulant that distinguishes it from other adjuvants. AS03 enhances immune responses by stimulating local immune cells and promoting antigen uptake and presentation. These mechanisms lead to a potent and broad antibody response, evidenced by memory B‐cell progressive maturation and somatic hypermutation, which enhances neutralization breadth over time [43].

In 2008, the European Medicines Agency authorized AS03‐adjuvanted influenza vaccines against H5N1 (Prepandrix; GSK) [44] and H1N1 (Pandemrix; GSK) [45], with the latter being widely used during the 2009 influenza H1N1 pandemic [46]. Post‐2009 pandemic surveillance highlighted increased narcolepsy incidence associated with Pandemrix vaccine, particularly in individuals with specific genetic profiles (HLA‐DQB1*06:02), suggesting an interplay of genetic factors and immune responses [47, 48]. Despite these concerns, many studies have consistently affirmed AS03 safety and efficacy in IC individuals, supporting its continued use against seasonal and pandemic influenza across diverse demographic profiles [48]. Furthermore, AS03, which is frequently used for vaccine development, has also been studied for potential use in SARS‐CoV‐2 vaccines [49]. The successful applications of AS03 highlight the potential role of tailored adjuvants in enhancing vaccine responses in IC individuals.

### 2.6 AS01_B_

Developed over 20 years ago, AS01_B_, another adjuvant system developed by GSK, utilizes a liposomal base to deliver two immunostimulants: MPL and QS‐21, the latter of which is a natural saponin compound with intrinsic lytic activity extracted from the Chilean soapbark tree (*Quillaja saponaria*) [50, 51]. While QS‐21 MOA has not been confirmed, QS‐21 increases cytotoxic T‐cell and T‐helper cell responses to the vaccine protein antigen and intensifies the humoral response [52]. Unlike oil‐in‐water emulsion‐based adjuvants such as AS02_B_ and AS02V, AS01_B_ enhances immune responses through the TLR activation by MPL, triggering cytokine‐mediated processes crucial for both innate and adaptive immunity, including maturation of T and B cells [52]. The combination of MPL and QS‐21, two well‐established adjuvant molecules, is synergistic, resulting in a more robust immune response.

SHINGRIX (GSK) combines varicella zoster virus glycoprotein E with AS01_B_ for the prevention of herpes zoster in older adults. This inactivated recombinant zoster vaccine (RZV) has shown efficacy in immunocompetent recipients, including those over 70 years of age. However, patients on higher doses of immunosuppression were excluded from initial efficacy trials (ZOE‐50 and ZOE‐70) [53]. Although RZV has demonstrated variable immunogenicity in the stimulation of humoral responses among patients with diffuse large B‐cell lymphoma, the cellular immune response crucial for varicella zoster virus immunity remains robust. Safety has also been confirmed in SOT and HCT recipients [54, 55, 56].

The AS01_B_ adjuvant system is being used in numerous investigational trials of HIV, malaria, and hepatitis B vaccines, demonstrating safety and considerable induction of humoral and cell‐mediated immune responses [52, 57, 58]. It has been extensively tested for safety and immunogenicity in IC individuals, including those living with HIV, hematologic malignancies, and oncologic conditions.

Current guidelines from the American Society of Transplantation (AST) recommend pre‐transplant immunization with two doses of RZV [59]. However, guidance on post‐transplant vaccination remains limited. While safety and immunogenicity were demonstrated in several studies [54–56, 60], studies assessing efficacy in disease prevention are ongoing. A randomized clinical trial involving autologous HCT recipients demonstrated 68% overall vaccine efficacy (VE), though efficacy was lower in post‐transplant patients ≥50 years of age [61]. A post hoc analysis conducted on the ZOE‐50 and ZOE‐70 trials reported 90.5% VE among participants with at least one potential immune‐mediated disease [62]. Those findings eventually led to FDA approval in July 2021 of RZV in those ≥18 years old with IC defined by diagnosis or treatment [63].

Concerns have been raised regarding the use of RZV in SOT recipients, because safety studies were conducted among patients on lower doses of immune suppression, who had a lower risk of graft rejection and were further away from their transplant [54]. Nonetheless, the theoretical potential for AS01_B_ to trigger graft rejection in SOT patients receiving RZV has not been systematically assessed. Combined moderate‐to‐high efficacy, immunogenicity, and safety in the overall IC population led the ACIP in January 2022 to recommend RZV in immunosuppressed patients ≥19 years of age, given the high morbidity and mortality associated with herpes zoster in this group [63].

### 2.7 Alhydroxiquim‐II

The coronavirus disease 2019 (COVID‐19) pandemic spurred the development of new vaccine adjuvants to ensure a sustained immune response against SARS‐CoV‐2. Alhydroxiquim‐II (AHQ‐II) was developed by ViroVax LLC of Lawrence, Kansas [64], with support from the National Institute of Allergy and Infectious Diseases (NIAID) Adjuvant Development Program. AHQ‐II, which comprises imidazoquinoline (IMQ) adsorbed onto alum [65], functions as a TLR7 and TLR8 agonist. Activation of TLR‐7 and TLR‐8 on APCs such as DCs and monocytes initiates a signaling cascade (e.g., NF‐κB and mitogen‐activated protein kinases [MAPKs]) that leads to the production of proinflammatory cytokines and type I interferons, promoting a Th1‐biased response and enhancing the activation of CD4⁺ and CD8⁺ T cells [66, 67]. This mechanism supports a robust cellular immune response, which is critical for antiviral defense [64, 65].

AHQ‐II is a key component of the SARS‐CoV‐2 inactivated virus vaccine, BBV152/COVAXIN, which was developed by Bharat Biotech in collaboration with the Indian Council of Medical Research (ICMR) and National Institute of Virology (NIV). After demonstrating efficacy against COVID‐19 and safety in clinical trials, including children [68, 69, 70], BBV152/COVAXIN received emergency use authorization from the World Health Organization (WHO) in November 2021 [71]. Although the WHO had provisionally recommended BBV152/COVAXIN for IC individuals [72], safety and efficacy data in this population were lacking. BBV152/COVAXIN immunogenicity was notably reduced in IC recipients [73]. Due to low effectiveness against viral variants of concern, the WHO suspended BBV152/COVAXIN use in April 2022 [74]. Therefore, further research is needed to determine the utility of AHQ‐II in IC populations.

### 2.8 Virosomes

Virosomes are sophisticated vaccine delivery systems that encapsulate vaccine antigens within a lipid bilayer decorated with viral envelope proteins such as influenza hemagglutinin, enabling effective direct or endosomal‐mediated antigen delivery into the cytosol of target cells [75, 76]. Mimicking natural viral infections, virosomes enhance immune responses without the potential risks of replication, making them particularly safe and effective.

Since their introduction in the 1990s, virosomes have been incorporated into several licensed vaccines, including Inflexal V (Crucell) for influenza and Epaxal (Crucell) for hepatitis A, demonstrating success across various populations [76, 77]. Safety and immunogenicity are well‐documented; for instance, Inflexal V induces strong immune responses with minimal side effects in IC individuals [77].

While virosome‐based candidate vaccines are in development for several common pathogens, including HIV, HPV, respiratory syncytial virus (RSV), and malaria [78], virosomes also serve as a promising platform for the development of tailored vaccination strategies in IC individuals, given favorable immunogenic properties and tolerability [79].

### 2.9 RC‐529

RC‐529, a synthetic lipid A mimetic, acts as a TLR4 agonist to stimulate both innate and adaptive immunity. Developed as a less toxic alternative to MPL, RC‐529 has been evaluated in vaccines targeting diseases such as hepatitis B and cancer [80, 81]. A clinical trial in Argentina demonstrated that a hepatitis B vaccine adjuvanted with RC‐529 was more immunogenic than its nonadjuvanted counterpart and was well‐tolerated, with transient local reactions. By Day 90, 99.2% of healthy adults who received the adjuvanted vaccine achieved seroprotection compared with 83.9% of those who received the standard vaccine [80].

Safety and immunogenicity of RC‐529 have not been specifically studied in IC hosts; however, its MOA indicates potential benefits for these individuals. RC‐529‐adjuvanted hepatitis B vaccine licensed in Argentina under the name “Supervax” (Dynavax) is no longer in use [82]. Further research is necessary to confirm the adjuvanticity of RC‐529 and expand its applications to other vaccines and IC populations.

## 3 Candidate Adjuvants

The field of vaccine adjuvants is rapidly evolving, with several promising products currently in development or clinical trial phases. These adjuvants could lead to advancements in vaccine immunogenicity and effectiveness, particularly for IC individuals. However, as candidates progress through clinical trials, it is important to monitor not only immunogenicity but also safety profiles, particularly in complex populations such as the immunocompromised, who may be prone to unusual and unpredictable adverse effects. Here, we briefly address novel pipeline adjuvants.

### 3.1 AS01 and AS02

The AS system of vaccine adjuvants is liposome‐based and used to enhance innate and adaptive responses against target pathogen antigens [83]. Both AS01 and AS02 have been utilized in malaria vaccines, and ongoing research is exploring their applicability to other infections, including RSV, non‐typable *Hemophilus influenzae, Moraxella catarrhalis*, HIV, *Mycobacterium tuberculosis*, and hepatitis B, in addition to being considered for cancer therapeutics [84, 85, 86, 87, 88].

Patients with renal insufficiency requiring dialysis have impaired immunity and suboptimal response to hepatitis B vaccination. In a Phase III clinical trial of immunogenicity and safety of an AS02‐adjuvanted hepatitis B vaccine in patients with end‐stage renal disease, Surquin et al. found enhanced and extended protection in patients who received three doses of AS02‐adjuvanted vaccine compared to the standard four‐dose AS04‐adjuvanted vaccine. Among those who received the AS02 containing vaccine, 77.0% and 93.6% achieved seroprotection at Months 2 and 12, respectively, after the initial dose, compared with 39.0% and 78.6%, respectively, in the control group [89].

The adjuvanted malaria vaccines RTS,S/AS01 (Mosquirix; GSK) and RTS,S/AS02 (GSK) have demonstrated improved immune responses and acceptable safety profiles compared with unadjuvanted vaccines. The RTS,S/AS01 formulation in particular improved induction of the T‐cell response and produced significantly higher *Plasmodium falciparum* circumsporozoite mean geometric antibody titers compared to RTS,S/AS02, illustrating the potential of AS01 to bolster immunogenicity of novel vaccines [57, 90–94].

### 3.2 Army Liposome Formulation

Army Liposome Formulation (ALF) adjuvants were developed at the Walter Reed Army Institute of Research and have been a pivotal innovation as a vaccine adjuvant that provides excellent safety and potency and could carry both military and civilian benefits [40, 95]. These adjuvants are liposomal formulations containing saturated phospholipids, cholesterol, and MPL. ALF adjuvants act as a vehicle for the antigen, promoting antigen uptake by immune cells and amplifying the immune response [96, 97]. ALF adjuvants have demonstrated a capacity to enhance both humoral and cellular immune responses in HIV and malaria vaccines [95, 97].

### 3.3 Lipid A in Stable Emulsion (GLA‐SE)

Glucopyranosyl lipid A, a synthetic derivative of MPL, in a stable oil‐in‐water emulsion (GLA‐SE) is another TLR4 agonist. It is being evaluated for use in vaccines targeting diseases such as influenza, tuberculosis, and malaria [98, 99, 100, 101]. GLA‐SE has also been used as a novel tumor vaccine adjuvant with the potential to augment the antitumor immune response in patients with resected melanoma [102]. GLA‐SE has shown promise in stimulating robust immune responses in both preclinical and early clinical trials.

### 3.4 Saponin Complexes (ISCOM, Matrix‐M)

Saponin‐based adjuvants, such as immune‐stimulating complexes (ISCOM) and Matrix‐M, form ring‐like micellar nanoparticles with saponins, cholesterol, and phospholipids that mimic the structure of viruses. This configuration stimulates robust humoral and cellular immune responses [103, 104]. Saponin complexes have been employed in vaccines targeting influenza, HIV, and HPV [105, 106, 107, 108]. Novavax's Matrix‐M adjuvant, for example, has been incorporated into the company's COVID‐19 vaccine candidate NVX‐CoV2373, highlighting the potential of saponin complexes to enhance immune responses against emerging infectious diseases [109]. The vaccine was authorized for emergency use by the US FDA in July 2022 under the trade name “Novavax COVID‑19 Vaccine, Adjuvanted” [110].

While NVX‐CoV2373 has shown safety and efficacy in the general population, data on its use in IC individuals remain limited. In a Phase III trial of NVX‐CoV2373, individuals with immunocompromising conditions were an exclusion criterion; however, participants with stable chronic medical conditions, including those living with HIV on effective antiretroviral therapy, were eligible for enrollment. The trial did not report outcomes specifically for the subgroup with chronic medical conditions, nor did it describe vaccine immunogenicity or efficacy in the context of immunosuppression [111]. Therefore, as NVX‐CoV2373 becomes more widely available, further research will be essential to evaluate its immunogenicity, safety, and clinical effectiveness in the setting of immunocompromise.

### 3.5 IC31

IC31 (Intercell, Vienna, Austria) is a synthetic adjuvant combining a derivative of a natural cationic antimicrobial peptide with a synthetic oligodeoxynucleotide (ODN1a). The peptide enhances antigen‐specific antibody production, while ODN1a acts as a TLR9 agonist that eventuates in a Th1‐type adaptive profile via stimulation of the innate response [112]. IC31 has demonstrated safety and efficacy in boosting immune responses in both preclinical and early clinical trials, including those targeting tuberculosis [113, 114, 115, 116]. Its ability to stimulate both innate and adaptive immunity makes it a promising candidate for a variety of vaccines.

### 3.6 Other Candidates

Several other novel adjuvant candidates are currently in various stages of preclinical and clinical development, with the potential to enhance vaccine immunogenicity and protect against a wide range of infectious diseases. These include ISA‐51 (Montanide; SEPPIC), a water‐in‐oil emulsion; flagellin, a TLR5 agonist; poly I:C and its derivatives, synthetic double‐stranded RNA analogs and TLR3 agonists; Vaxfectin (Vical), a cationic liposome; the EGVac system, a bacterial polysaccharide/DNA adjuvant; and PIKA (Liaoning Yisheng Biological Pharmaceutical Co.), another double‐stranded RNA analog and TLR3 ligand. These novel adjuvants have demonstrated robust cellular and humoral immune responses in clinical trials, with some showing potential for specific populations, such as the elderly or those receiving mucosal vaccines. Although data on their use in IC individuals are limited, immunostimulatory properties of these adjuvants suggest utility in enhancing vaccine efficacy in this population [117, 118]. Continued research will help refine safety and efficacy profiles of next‐generation adjuvants.

## 4 Safety of Adjuvants

Adjuvants have been used for over a century to enhance vaccine immunogenicity and increase effectiveness [119]. Despite extensive research, only a limited number of adjuvants have been approved for human use due to safety concerns [34, 119]. Adjuvant safety depends not only on the adjuvant itself but also on the manufacturing process and the combination of adjuvant type and vaccine antigen [120]. According to the CDC, adjuvanted vaccines have been associated with an increased frequency of local reactions (redness, swelling, and pain at the injection site) and systemic reactions (fever, chills, and body aches) [12].

While some safety concerns associated with new adjuvant development can be identified during Phase I–III trials, unpredictable and rare complications may appear in specific populations or may not be discovered until widespread introduction following vaccine approval [120]. For example, several cases of Bell's palsy were identified in Switzerland after the introduction of a novel intranasal influenza vaccine that contained *Escherichia coli* heat‐labile toxin as a mucosal adjuvant. These findings have slowed the development of adjuvanted intranasal vaccines [121].

As previously noted in the discussion of the AS03 adjuvant, vaccine‐associated narcolepsy has caused controversy and unease in the vaccine community [122]. Some epidemiological studies linked the increase in narcolepsy among children in specific geographical areas to the use of adjuvanted influenza vaccine, Pandemrix (GSK), specifically among those with the HLA‐DQB1*06:02 allele [47]. It is argued that this reported association might be due to the use of the well‐established AS03 adjuvant, although some animal model findings indicate that intranasal instillation of the H1N1 virus itself has the potential to cause narcoleptic‐like sleep disruption [120, 122, 123]. Other studies found no association between this vaccine and narcolepsy in children [124]. These inconsistent findings highlight the challenges of establishing causality in vaccine safety studies and illustrate potential interplay between vaccine components and host genetic susceptibility. Thus, post‐marketing vaccine safety surveillance is critical.

For IC individuals, the timing, type, and safety of adjuvanted vaccines are very important considerations [125]. One prominent concern is an increased risk of local and systemic reactions, with a higher frequency of Grade 3 reactions in IC individuals (local reactions: 10.7%–14.2%; systemic reactions: 9.9%–22.3%) after the administration of the adjuvanted RZV, SHINGRIX, compared to placebo group (local reactions: 0.0%–0.3%; systemic reactions: 6.0%–15.5%) [63, 126]. These data, synthesized from multiple clinical reports, point to the significant reactogenicity of SHINGRIX compared with placebo; however, direct comparisons between IC and immunocompetent vaccinees are lacking. Although immunosuppression may theoretically reduce reactogenicity for some vaccines, potent immunostimulation by adjuvants such as AS01B can induce considerable local and systemic reactions even in IC individuals. Therefore, careful consideration of potential reactogenicity remains crucial despite the underlying immunodeficiency.

Adjuvants stimulate the immune system to produce a stronger and more durable response, leading to theoretical concerns that adjuvanted vaccines could increase the risk of organ rejection or graft‐versus‐host disease in IC individuals. However, studies assessing outcomes of adjuvanted influenza vaccination in SOT recipients have not revealed sufficient clinical evidence of vaccine‐mediated rejection [127]. In general, heterogeneity within the IC population presents a significant barrier to uniform evaluation of adjuvant efficacy and safety. Both parameters depend on the type of immunodeficiency (e.g., primary or secondary immunodeficiency), specific condition (e.g., solid organ or hematopoietic cell transplantation, HIV, or autoimmunity), type and intensity of immunosuppressive therapy, and age of the vaccinee. Data derived from one IC subpopulation does not necessarily apply to another. Significant feasibility hurdles and lag in adjuvant research relative to other areas in vaccinology have impeded direct comparative studies across different IC subpopulations, leading to knowledge gaps about adjuvant potential to stimulate immunopathology in grafted or native tissue. Therefore, data should be extrapolated cautiously, and future research should prioritize adjuvant evaluation in well‐defined subpopulations, including children.

Autoimmune/inflammatory syndrome induced by adjuvants (ASIA) was a term introduced in 2011 to summarize the spectrum of immune‐mediated diseases triggered by adjuvants [128]. A 2019 review explored a potential link between aluminum‐based adjuvants and myalgic encephalomyelitis/chronic fatigue syndrome [129]. While theoretical risks exist for potential immune‐mediated diseases associated with adjuvanted vaccines in IC individuals, a review of six trials involving RZV in older IC adults did not find an association between vaccine receipt and the onset or exacerbation of potential immune‐mediated diseases [130].

While available evidence does not suggest a heightened risk of adverse outcomes in IC individuals from vaccines containing adjuvants, several factors should be carefully weighed when assessing adjuvant safety, including adjuvant type, antigen–adjuvant combination, manufacturing process, and the particular type of immunocompromise. These considerations will grow in importance as the number and diversity of vaccine adjuvants increase alongside a growing IC population relying on adjuvanted vaccines for protection.

## 5 Conclusion

Vaccine adjuvants are pivotal in enhancing vaccine immunogenicity and efficacy, particularly among IC individuals who respond suboptimally to traditional vaccines compared to their healthy counterparts. Recent advancements have ushered in a variety of novel adjuvants such as MF59 and AS04, which have demonstrated the capacity to potentiate immune responses effectively. These adjuvants, having shown promising results in improving protection against various infectious diseases, are now integral to several licensed vaccines. However, additional clinical research is required to ensure safety and effectiveness in these groups. Future investigation of new candidate adjuvants should likewise prioritize safety and immunogenicity in IC individuals, recognizing the need for data specific to well‐defined IC subpopulations. Meanwhile, clinicians managing IC individuals should prioritize use of currently recommended adjuvanted vaccines (e.g., SHINGRIX, HEPLISAV‐B, and adjuvanted seasonal influenza vaccines) according to established guidelines while remaining cognizant of efficacy and safety considerations discussed herein.

Our expanding understanding of immune mechanisms and adjuvant actions continues to open avenues for more targeted and effective vaccine strategies. Leveraging systems biology to understand individual immune responses and incorporating advanced formulation technologies can drive the development of next‐generation vaccine adjuvants. Such strategic progress is crucial for creating vaccines that confer optimal protection for all, including those with compromised immune systems.

From a public health policy standpoint, findings reviewed here highlight several priority areas. First, there is a clear need for policy support to incentivize and fund research focused on adjuvant efficacy and safety within and between well‐defined IC subpopulations. Second, it is essential to harmonize this research with robust post‐marketing surveillance systems capable of detecting rare adverse events in IC groups. Finally, the timely development of evidence‐based clinical guidelines (such as those from the ACIP and professional associations) that explicitly address adjuvant use in specific IC subpopulations and promote access to these products is a critical policy consideration for public health, especially as it pertains to vaccines against emerging infections.

The field of vaccine adjuvants is rapidly evolving, with a robust pipeline of candidates showing significant potential. As we confront established, emerging, and re‐emerging infectious diseases, prioritizing adjuvant development tailored to the unique needs of the expanding IC population is imperative. Enhanced collaboration among academia, industry, and regulatory bodies is essential to accelerate the clinical translation of novel adjuvants, ultimately fostering improved global health outcomes and diminishing the infectious disease burden universally.

## Author Contributions

**Haya Hayek**: conceptualization; investigation; writing ‐ original draft; writing ‐ review and editing. **Lana Hasan**: investigation; writing ‐ original draft; writing ‐ review and editing. **Justin Z. Amarin**: investigation; writing ‐ original draft; writing ‐ review and editing. **Yasmeen Z. Qwaider**: investigation; writing ‐ original draft; writing ‐ review and editing. **Olla Hamdan**: investigation; writing ‐ original draft; writing ‐ review and editing. **Wanderson Rezende**: writing ‐ review and editing. **Kevin C. Dee**: investigation; writing ‐ original draft; writing ‐ review and editing. **James D. Chappell**: investigation; supervision; writing ‐ review and editing. **Natasha B. Halasa**: conceptualization; investigation; supervision; writing ‐ review and editing.

## Disclosure

J.D.C. reports research support from Merck. N.B.H. received grant support from Sanofi and Quidel, reports a current investigator‐initiated grant from Merck, and serves on an advisory board for CSL Seqirus. The authors report no other disclosures.

## Data Availability

No original data are associated with this article.
